# In-Vivo Biophoton Emission, Physiological and Oxidative Responses of Biostimulant-Treated Winter Wheat (*Triticum eastivum* L.) as Seed Priming Possibility, for Heat Stress Alleviation

**DOI:** 10.3390/plants11050640

**Published:** 2022-02-26

**Authors:** Ildikó Jócsák, Henrik Gyalog, Richárd Hoffmann, Katalin Somfalvi-Tóth

**Affiliations:** Department of Agronomy, Institute of Agronomy, Kaposvár Campus, Hungarian University of Agriculture and Life Sciences, Guba Sándor Street 40, H-7400 Kaposvár, Hungary; gyahen@gmail.com (H.G.); hoffmann.richard@uni-mate.hu (R.H.); somfalvi-toth.katalin@uni-mate.hu (K.S.-T.)

**Keywords:** wheat, heat stress, biophoton emission, biostimulant, lipid oxidation, antioxidant capacity, non-invasive stress detection

## Abstract

High temperature induces oxidative processes in wheat, the alleviation of which is promising using biostimulants. Priming has been used for enhancing stress tolerance of seedlings. However, the usage of biostimulants for priming is an unexplored area under either normal or stress conditions. Therefore, the aim of our study was to evaluate the heat stress alleviation capability of differentially applied biostimulant treatments on wheat seedlings. The investigation included stress parameters (fresh/dry weight ratio, chlorophyll content estimation, antioxidant capacity and lipid oxidation) combined with biophoton emission measurement, since with this latter non-invasive technique, it is possible to measure and elucidate in vivo stress conditions in real-time using lipid oxidation-related photon emissions. We confirmed that a single biostimulant pretreatment increased antioxidant capacity and decreased biophoton release and lipid oxidation, indicating the reduction of the harmful effects of heat stress. Therefore, biophoton emission proved to be suitable for detecting and imaging the effects of heat stress on wheat seedlings for the first time. Two-way analysis of variance (ANOVA) revealed that biostimulant (*p* = 4.01 × 10^−7^) treatments, temperature (*p* = 9.07 × 10^−8^), and the interaction of the two factors (*p* = 2.07 × 10^−5^) had a significant effect on the overall count per second values of biophoton emission, predicting more efficient biostimulant utilization practices, even for seed priming purposes.

## 1. Introduction

Wheat is one of the most important cereals [1]. Its applications are very diverse, ranging from food ingredients to feeds; additionally, due to its high adaptive capability, wheat is in cultivation worldwide [2] with several varieties selected recently or very ancient [3]. The main barrier to wheat production lies in changing climatic conditions, which have manifested in more frequently occurring extremities, the most important of which is increasing temperature; in particular, extreme temperature events impair the predictability of atmospheric processes during the autumn [4]. Many farmers prefer sowing wheat in the second half of September for better and safer autumn bushing, increasing the risk of exposure to high temperatures especially in the case of shallow or uneven sowing. Lei et al. [5] state that a temperature of 20–25 °C is optimal for germination; however, temperatures above 35 °C have a detrimental effect on germination processes.

High temperature stress causes oxidative stress in wheat, altering the photosynthetic and respiration-related processes and causing dysfunctions of the electron transport chains [6]. The longer the heat stress lasts, the more negative the effects it has on the physiological processes leading to increased transpiration, but at the same time decreasing stomatal conductance, resulting in a decrease in net assimilation [7]. Heat stress also causes a significant decrease in chlorophyll content [8] and photosynthetic and respiratory function, resulting in the formation of free radicals. Free radicals are oxygen-, nitrogen-, sulfur- or carbon-centered molecules formed due to unfavorable external effects [8]. Free radicals have high reactivity and short lifespans, and may alter the structure and function of many molecules including membranes, leading to altered physiological processes [9]. Reactive oxygen species (ROS) are a group of free radicals that have high affinity for adjacent compounds due to their unpaired electrons, leading to oxidative modifications of proteins, DNA damage and lipid peroxidation; the primary consequence of the latter is the formation of hydroperoxides, which are responsible for damaging other molecules. Moreover, ROS induce membrane disintegration followed by the release of specific lysosomal enzymes, causing tissue damage. Damage to proteins reduces the activity of enzymes, while damage to nucleic acids carrying genetic information results in mutagenic, carcinogenic or cell-destroying effects [10]. Non-invasive stress detection and imaging techniques are highly important in plant research, because they enable the physiological responses of plants to be objectively identified and create the opportunity to conduct measurements on the same plant individual later, in a further developmental stage, or in stress conditions [11,12].

Biophoton emission (BPE) measurement is an emerging non-invasive in vivo imaging technique [13]. Plants emit photons; this occurs primarily during the oxidation of cell constituents, such as lipids, proteins and nucleic acids, bringing about the development of excited species, which then go through a transition phase, then return to their ground state emitting photons in the meantime [13,14] from various plant parts and tissues: chloroplasts [15], leaves [16,17,18], an aqueous extract of soybeans [19], and also in azuki bean and soybean seedlings [20,21]. It has been observed that biophoton release during lipid peroxidation shows intense activity, thus allowing accurate traceability of the plant stress response and oxidative damage. The emitted photons can be detected and measured in real-time; thus, it is possible to gain insight into the metabolic activity and oxidative state of the plant.

Stress-induced biophoton emissions occur consequently with the formation and transformation of reactive oxygen species (ROS) during certain enzymatic processes of homeostatic or stress-induced mechanisms of electron transport chains [18,22] and can be analyzed qualitatively and quantitatively based on images and acquired data, thus allowing detection of the oxidative damage caused by various stress factors even before the appearance of visible symptoms [18].

Along with the reduced use of synthetic chemicals in agricultural practices, climate change poses severe challenges for plant cultivation. These two factors have prompted a reconsideration of the possibilities for application of the active ingredients in the products in current use. Besides the non-invasive stress detection methods mentioned above, seeking effective stress alleviation possibilities is also a major task and challenge of plant stress research. The oxidative consequences of heat stress can be prevented and reduced with the help of antioxidant molecules [23]. Antioxidants include vitamins, amino acids, enzymes, plant metabolites and minerals [23], and biostimulant products are extracts obtained from organic substances containing biologically active ingredients that exert antioxidant properties [24]. Their most common constituents are mineral elements, humic acids, vitamins, amino acids, hormones, polysaccharides and oligosaccharides [25,26,27]. They may also contain hormones produced by algae, such as cytokines, auxins, and gibberellins [27]. Their beneficial effects are usually effected via foliar application, leading to an increase in photosynthetic activity and tolerance to abiotic stressors and recovery from metabolic imbalances in plants [27]. The need for more efficient and economical utilization of agricultural inputs gave rise to the development of seed pretreatment technologies, such as priming. Priming of seeds includes different pretreatments of seeds before sowing, such as presoaking in water, called hydropriming, or treatment with biologically active substances that may improve seedling and plant characteristics [28].

Seed priming has been used not only to enable germ-free seeds to sprout, but recently also for enhancing the growth and developmental properties [20], as well as stress tolerance, of seedlings [28,29].

Since seed germination involves several metabolic processes, such as antioxidant pathways, deoxyribonucleic acid (DNA) replication responses, greater adenosine triphosphate (ATP) release, and the biosynthesis of membranes and proteins [28], the preapplication of biostimulants for seed priming may be beneficial in alleviating heat stress due to their antioxidant properties, and/or amino acid content.

Based on the studied literature, limited data are available on biophoton emission in wheat plants exposed to heat stress [28], and the effect of seed pretreatment [29,30] with biostimulant compounds under ideal and elevated temperatures in wheat is still an unexplored area. Therefore, the aim of our study was to evaluate the heat stress alleviation capability of differentially applied biostimulatory treatments in wheat seedlings. The investigation was conducted by measuring and analyzing changes in conventional stress parameters (fresh/dry weight ratio, SPAD index, antioxidant capacity and lipid oxidation) along with lipid oxidation-related biophoton emissions, using non-invasive stress imaging techniques in order to gain insight into the applicability of biostimulant pretreatment to the alleviation of heat stress in wheat.

## 2. Results 

### 2.1. Estimation of Fresh/Dry Weight Ratio (FDWR) and Chlorophyll Content (SPAD) in Wheat Seedlings

**Figure 1:**
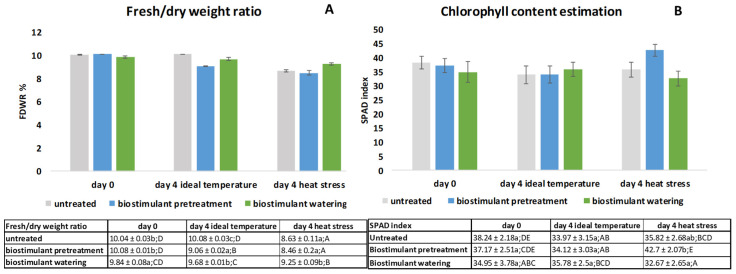
(**A**) Fresh/dry weight ratios (*n* = 3) and (**B**) chlorophyll content estimation (*n* = 10) of wheat leaves grown for 4 days under ideal and heat-stressed conditions at the same time subjected to biostimulant pretreatment or biostimulant watering. The same letters represent non-significant differences within each treatment (*p* ˂ 0.05). Upper-case letters refer to the time effect, whereas lower-case letters refer to the effect of biostimulant treatment within one sampling occasion.

The fresh/dry weight ratios of samples are presented in Figure 1A. The FDWRs of 10- day-old seedlings were similar in UT and BPT samples with significantly higher values compared to BW samples, as confirmed by one-way ANOVA (*p* < 0.05) and Duncan post hoc test. After 4 days of ideal temperature conditions, BPT samples showed the lowest FDWR (Figure 1A), and the FDWR values were significantly different from each other as indicated by the statistical analysis. However, 4 days of HS increased FDWR significantly in the BW samples (Figure 1A), for a 107% increase compared to UT and 109% compared to BPT samples.

When analyzed according to the effect of time on FDWR, the highest values were obtained for the first sampling day, then after 4 days of ideal temperature, BPE samples had the lowest, and UT samples the highest, FDWR values. All three sample types were significantly different according to the results of one-way ANOVA (*p* < 0.05) and Duncan post hoc test.

According to the results of two-way ANOVA, biostimulant treatments (*p* = 3.5 × 10^−4^), temperature (*p* = 2.05 × 10^−10^), and their interaction (*p* = 3.81 × 10^−5^) had a significant effect on FDWR (Figure 1A). 

The chlorophyll content estimation was quantified via SPAD index, and the results are presented in Figure 1B. On the first sampling day, UT and BW samples were different as confirmed by one-way ANOVA (*p* < 0.05) and Duncan post hoc test. Four days under ideal temperature did not induce any difference among the sample types. However, 4 days of HS resulted in increased chlorophyll content in BPT, by 19% compared to that in the UT samples and by 31% compared to that in BW seedlings (Figure 1B). 

When analyzed according to the effect of time on the SPAD index, the highest value was obtained under HS conditions for BPT samples according to the results of one-way ANOVA (*p* < 0.05) and Duncan post hoc test.

Two-way ANOVA analysis showed that biostimulant treatments (*p* = 1.05 × 10^−3^), temperature (*p* = 1.8 × 10^−3^), and their interaction (*p* = 4.15 × 10^−5^) all had a significant effect on chlorophyll content estimates (Figure 1B).

### 2.2. Analyses of Antioxidant Capacity (FRAP) Results Based on Iron Reducing Ability and Lipid Oxidation Based on MDA Quantification

**Figure 2:**
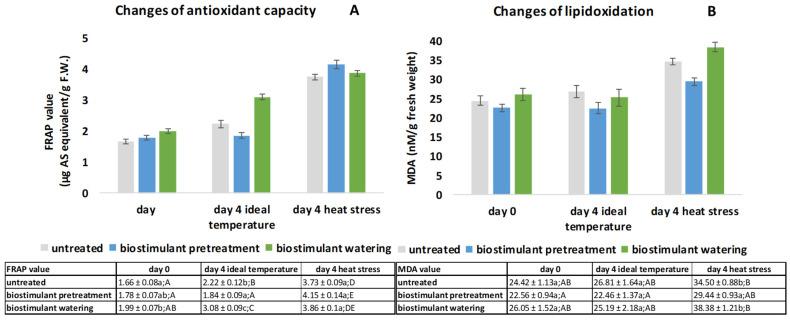
Changes in (**A**) ferric reducing antioxidant capacity and (**B**) lipid oxidation of wheat (*Triticum aestivum* L.) seedlings. Mean values (*n* = 3) and standard deviations are presented. The same letters represent non-significant differences within each treatment, analyzed with one-way ANOVA (*p* ˂ 0.05) with Duncan post hoc test. Upper-case letters refer to the time effect, whereas lower-case letters refer to the effect of biostimulant treatment within one sampling occasion.

Antioxidant capacity. The ferric reducing capacity of the wheat seedlings increased during the 4 days of the investigation under both ideal and HS conditions. On day 1, results of the Duncan test revealed a significant increase in the FRAP value of BW samples compared to that of the UT samples (Figure 2A). Having spent 4 days under ideal temperature conditions, the values for the UT and BW samples continued to rise, whereas the BPT samples did not show changes. However, 5 days of HS increased the FRAP values of all treatments by 68 % for UT (from 2.22 to 3.73 µg/AS equivalent/g F.W.), by 225% for BPT (from 1.84 to 4.15 µg/AS equivalent/g F.W.) and by 25 % for BW (from 3.08 to 3.86 µg/AS equivalent/g F.W.) seedlings, respectively (Figure 2A). 

After 4 days spent under ideal temperature, the FRAP values of the wheat seedlings were significantly different among the three samples, confirmed by one-way ANOVA (*p* < 0.05) and Duncan post hoc test, and the lowest value belonged to BPT samples. HS resulted in the highest FRAP values among all the investigated samples; the BPT sample had the highest values during the whole duration of the experiment, increasing to 4.15 (µg/AS equivalent/g F.W.) from 1.84 (µg/AS equivalent/g F.W.) for a 225% increase. 

Two-way ANOVA analysis showed that the biostimulant treatments (*p* = 1.76 × 10^−4^), temperature (*p* = 1.9 × 10^−9^), and their interaction (*p* = 1.14 × 10^−5^) all had a significant effect on the antioxidant capacity of wheat samples (Figure 2A).

Lipid oxidation (MDA). According to the results of the Duncan post hoc test, the rate of lipid oxidation did not change by number of sampling days, except for 4 days of HS treatment, which led to a decrease in lipid oxidation by 13% in BPT samples, 14.1% in UT samples, and 14.7% in BW samples (Figure 2B). The highest concentration of malondialdehyde was measured in BW plants on day 4 of HS, and the lowest MDA values were observed in BPT seedlings on all 3 sampling occasions. 

Two-way ANOVA analysis revealed that lipid oxidation was not affected by biostimulant treatments (*p* = 9.26 × 10^−3^), but temperature (*p* = 1.9 × 10^−9^) and the interaction of the two factors (*p* = 1.53 × 10^−3^) had a significant effect on the lipid oxidation of wheat samples (Figure 2B).

### 2.3. Biophoton Emission Measurement Results and Their Evaluation

**Figure 3:**
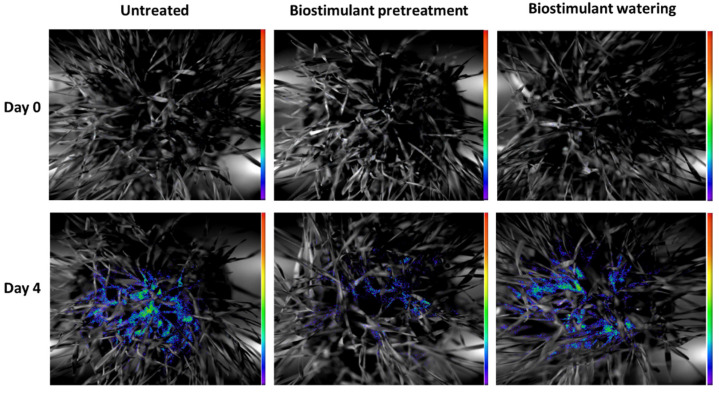
Images of the biophoton emission signals of 15-day-old heat-stressed wheat seedlings on day 0, as the starting point of HS, and on day 4. The intensity color bar shows signal intensity detected by the equipment and converted into a scale of pseudocolor intensity via the software.

**Figure 4:**
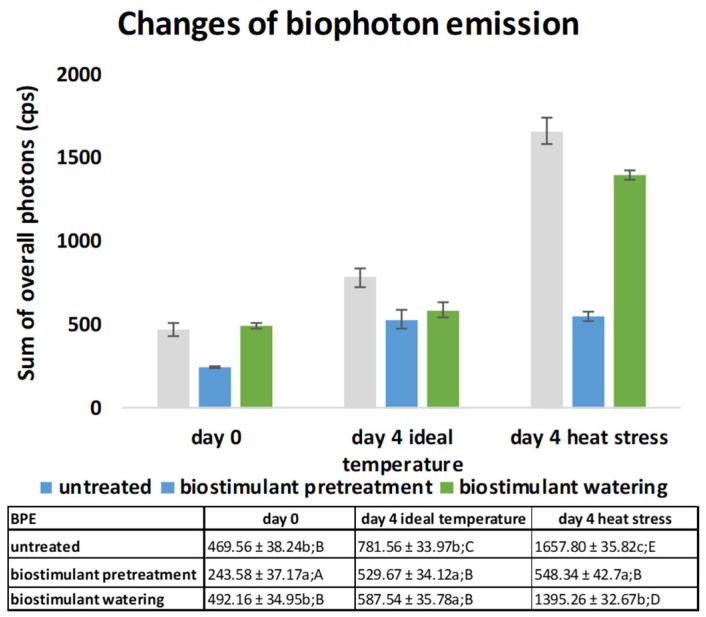
Sum of overall count per second values (cps) of wheat seedlings; mean values (*n* = 3) and standard deviations are presented. The same letters represent non-significant difference within each treatment, analyzed with one-way ANOVA (*p* ˂ 0.05) with Duncan post hoc test. Upper-case letters refer to time effect, whereas lower-case letters letter refer to the effect of biostimulant treatment within one sampling occasion.

The biophoton emission results obtained in our experiments are presented in Figure 3 and Figure 4. Figure 4 provides insight into the rate and induction of BPE signals from differentially applied biostimulant treatments in heat-stressed wheat seedling samples on day 1, the starting point of HS, and on day 4. According to the pseudocolor intensity, on day 1 the BPE signals were lower compared to those observed on day 4. Moreover, 4 days of heat stress resulted in increased signals for UT, BPT and BW seedlings, and the intensities indicated that BPT did not result in BPE signal-inducing effects as strong as those observed in the UT and BW samples (Figure 3).

As a next step of data analysis of biophoton data, the acquired signal intensities were collected as the sum of overall photons during 5 minutes of biophoton emission measurement. This approach not only enabled the imaging of the effects of the treatments, but also provided a useful parameter for precise quantitative analysis by integrating the data acquired per treatments. The results of the sum of overall count per-second values of photon emission are presented in Figure 4.

The BPE signals showed an increase from day 0 to day 4 in the UT samples under both temperature conditions, and by day 4, the BPE values had increased from 469.56 to 1657.81, for a 353% increase. This increase was lower in the value for the BPT samples, which was 243.58 on day 1 and increased to 548.34 on day 4. However, 4 days of heat stress did not elevate the BPE signals of BPT samples. This was not the case for BW samples, which showed a significant increase from 492.16 on day 1 to 1395.26 on day 4 of HS, for a 283.5% gain.

Two-way ANOVA analysis revealed that BPE was affected significantly by all of the treatments and sample types. Biostimulant (*p* = 4.01 × 10^−7^) treatments, temperature (*p* = 9.07 × 10^−8^), and the interaction of these two factors (*p* = 2.07 × 10^−5^) had a significant effect on the sum of overall count per second values of biophoton emissions in the wheat samples (Figure 4).

## 3. Discussion

In order to evaluate the growth and developmental processes-related stress alleviating capability of the biostimulant applied differentially, certain stress indicator physical parameters were determined, such as fresh/dry weight ratio of wheat leaves along with chlorophyll content estimation via SPAD index (Figure 1A,B). The results of these investigations revealed that both heat stress and biostimulant application affected the growth, as indicated by the changes in FDWR, and also the photosynthetic apparatus via the changes in chlorophyll content, depicted by the SPAD index. The FDWR reached by BPT samples was a result of higher dry matter content, showing the beneficial effect of biostimulatory pretreatment on wheat seedlings grown at ideal temperatures, as statistically confirmed by an increase in dry matter accumulation. Although both the drying of plants induced by HS and the effect of BPT treatment caused dry matter accumulation, leading to a decrease in FDWR, as confirmed by the significant effect of the combination of the two factors, heat stress (HS) and biostimulant treatment, the decreased FDWR of the BPT samples under ideal temperature conditions indicated the positive effect of the biostimulant treatment (Figure 1A). Our results are consistent with those of Van Oosten et al. [31], who found that the use of a biostimulant increased dry matter in spinach, lettuce, and wheat leaves in both normal and stressed states [31]. The results of chlorophyll content estimation via the measurement of the SPAD index did not show alterations under ideal conditions, but the biostimulant pretreatment significantly enhanced the SPAD index compared to both untreated and biostimulant watered seedlings under heat-stressed conditions. This indicated the enhancing effect of the biostimulant pretreatment. The results are in line with the works of Barutçular et al. [32], who stated that a decline in chlorophyll content was a sign of aging processes, similarly to stressful conditions generated by high temperature [33]. The SPAD index changes observed in our study (Figure 1B) are consistent with the work of Pantoja-Benavides et al. [34], who found that biostimulatory treatment increased chlorophyll content in the leaves of rice plants, thus positively contributing to heat stress tolerance.

Besides the negative effects of heat on morphological and physiological parameters, elevated temperature affects the membrane integrity of wheat, inducing ROS formation and triggering the antioxidative defense system of plants [35]. In addition to antioxidant enzymes, non-enzymatic antioxidants that are low molecular-weight compounds, such as ascorbic acid, carotenoids, tocopherol, polyphenols, polyamines, flavonoids, hormonal compounds (melatonin, serotonin) and amino acids, are capable of eliminating ROS without enzymatic reactions [36], and despite their small presence, greatly inhibit the oxidation of substrates [37], and are possible to characterize by the measurement of ferric reducing antioxidant capacity, i.e., FRAP [38]. Nevertheless, the measurement of lipid oxidation infers the magnitude of the concentration of unstable lipid peroxides with rapid degradation. One of the by-products is malondialdehyde, which can be used to characterize cell membrane damage in the case of heat stress due to lipid peroxidation [39]. In our studies, we found that HS affected both FRAP and MDA in an increasing way (Figure 2A,B). Similarly to our results, Miller et al. [40] found that heat stress increased MDA content in leaf by 27–58% during seedling development. Contrarily, we found that biostimulant pretreatment alleviated the negative effects: after 4 days of heat stress, the BPT samples had lower FRAP and MDA values, indicating that in these samples the activation of the antioxidant system was not necessary. 

Both the results of antioxidant capacity and lipid oxidation measurements showed similarities to those of Ali et al. [41], whose results indicated that seed priming with compounds with a biostimulatory role (garlic extract, methyl jasmonate and salicylic acid) significantly increased the activities of antioxidant enzymes such as superoxide dismutase, catalase and peroxidase and altered the level of lipid oxidation [41]. Our biostimulatory pretreatment studies also showed similar changes in these parameters with increased FRAP and MDA values.

According to the overall results of stress analytical parameters, the stress alleviating property of BST was manifested by the changes in FRAP (Figure 2A) and MDA (Figure 2B) values. Without HS, BPT plants showed low initial antioxidant capacity that, however, increased in the HS samples, maintaining low MDA values as a sign of induced enhanced stress tolerance. This is in agreement with findings from recent research that biostimulant spraying promotes better nutrient and water uptake and increases the level of stress tolerance by accelerating life processes [31], since our results imply that even one application on the seeds may have similar beneficial effects on seedling growth.

Their beneficial effects are usually manifested during application to the foliage, with an increase in photosynthetic activity as well as tolerance to abiotic stressors. They are suitable for conditioning and regenerating both the soil and the plant. Through their plant hormone content, they accelerate plant metabolism, thus enabling plants to survive the damage caused by stressors [31].

Finally, as a relatively new approach of in vivo imaging techniques in plant stress detection and characterization, biophoton emission was measured and quantified. Photon emission from plant cells is a phenomenon that can be detected and measured in real-time, which provides information on the oxidative state of the tissue [14,15,23], as the lower the biophoton emissions, the healthier the condition of the plant.

BPE results showed an increase during the 4 days of our investigations in UT, BPT, and BW samples as well, most likely as a consequence of normal seedling growth processes; however, BPE increased drastically due to HS in the UT and BW samples, but remained on a relatively low level in BPT samples (Figure 3 and Figure 4). This is in accordance with the lipid oxidation results of this investigation. According to the rate and intensity of the changes of biophoton emission, it can be inferred that it was the most sensitive parameter among all the measured stress indicators of heat stress along with the alleviating nature of biostimulant pretreatment. However, the results indicated that biostimulant watering also alleviated heat stress, as it was possible to detect and follow via pseudocolor intensities of the images acquired by BPE measurements, and, moreover, from the sum of overall BPE values of the samples. 

Besides the evident changes in growth, chlorophyll content estimated by SPAD, antioxidative capacity, and lipid oxidation, the most sensitive parameter of the investigation was biophoton emission. All of these measurements revealed, for the first time, the stress alleviating capability of the applied biostimulant, especially in when it was used as a priming pretreatment. This suggests future applications for biostimulants for seed priming purposes with the goals of both growth enhancement and heat stress alleviation [42]. When an organism develops under high-temperature conditions, heat-stressed protein denaturation and degradation of membrane structures are common. In addition, the presence of reactive oxygen species results in an increase in antioxidant enzyme activity and antioxidant capacity [42,43], as shown by an increase in biophoton emission in azuki beans exposed to heat shock. Their results are in line with our studies, as our results showed that biostimulatory pretreatment significantly increased antioxidant capacity, while keeping lipid oxidation levels low, as observed in BPT samples after 4 days of heat stress.

The current work proved that biophoton emission measurement is suitable for detecting the effects of heat stress on wheat seedlings; furthermore, we also confirmed that the use of biostimulants can reduce the harmful effects of ROS during heat stress, and this process was visualized for the first time by biophoton emission studies. Plants treated with biostimulants had higher antioxidant capacity and dry matter content during heat stress compared to the untreated group, as well as a significant decrease in biophoton release, indicating low lipid oxidation levels.

All of this suggests that the stress-reducing effects and effectiveness of a single biostimulant pretreatment outweigh the effects of biostimulant irrigation, which may predict the development of a more efficient and cost-effective method of biostimulant use.

## 4. Materials and Methods

### 4.1. Sowing and Germination 

The seeds were sown into 19 cm diameter pots and were placed in a climate chamber (Pol-Eco Apartura KK 1450, Kokoszycka 172C, 44-300 Wodzisław Śląski, Poland), with the following settings:

- day parameters: 22 °C [1]; 120 μM m^−2^ s^−1^; 16 h
- night parameters: 16 °C; 0 μM m^−2^ s^−1^; 8 h

Eighty seeds were sown in each pot in FLORIMO™ soil (consisting of peat, humus, manure, clay and fertilizers); pH 6.4 ± 0.5; density: 0.97 ± 0.1; dry matter (m/m%): 15.0; organic matter (m/m%): 70.0; N (m/m%): 1.0; P (m/m%): P_2_O_5_ 0.1, K_2_O (m/m%): 0.3. 

**Figure 5:**
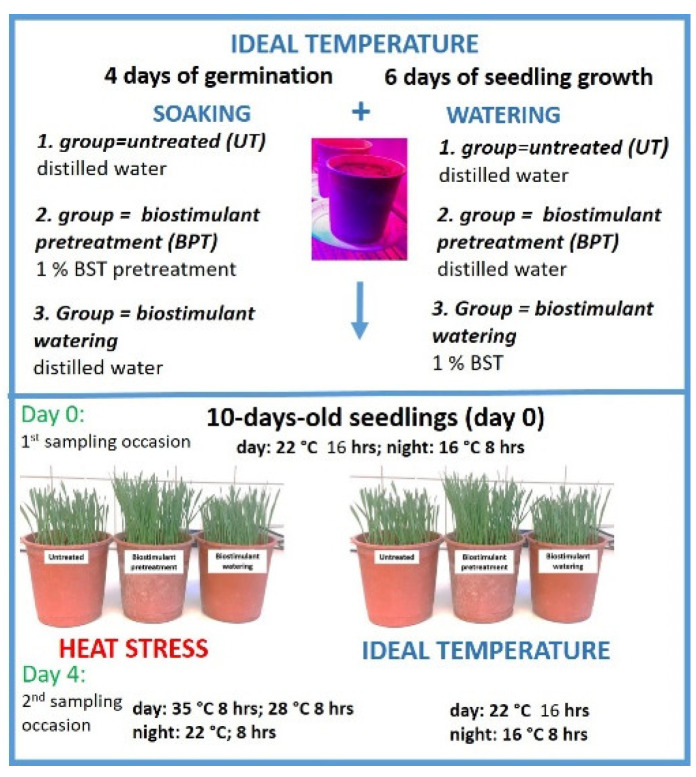
Plant growing, temperature conditions and biostimulant treatments.

The pots were divided into three groups and watered with only distilled water, or 1% biostimulant as indicated below (Figure 5). 

### 4.2. Biostimulant Treatments

1. Untreated (UT): the first group included the untreated seedlings that did not receive biostimulatory treatment. The watering of UT plants was carried out with distilled water.
2. Biostimulant pretreatment (BPT): the seedlings in the second group were pretreated with the biostimulant as indicated above, but after sowing and 4 days of germination, they were only watered with distilled water.
3. Biostimulant watering (BW): the seedlings in the third group were exposed to biostimulant watering. The germination of these seedlings was carried out in distilled water overnight, while subsequently, after 4 days of germination they received a watering of 1% biostimulant (Figure 5).

The composition of the commercially available biostimulant: seaweed extracts, vegetable oils, mineral oils, essential oils and herbal extracts in proportions specified by the manufacturer: pH 8.5 ± 0.5 (in 10 % water suspension); density: 0.97 ± 0.1; dry matter (m/m%): 15.0; organic matter (m/m%): 30.0; N (m/m%): 0.2; K_2_O (m/m%): 0.3. 

### 4.3. Growing Period of Wheat Seedlings

After the 4 days of germination under the conditions described above, the growing period of UT, BPT and BW plants lasted for another 6 days before the temperature conditions described below for Experiment I and II were initiated (Figure 5).

### 4.4. Experiment I: The Ideal Temperature

Experiment I started on 10-day-old seedlings, and instrumental and analytical measurements were performed on days 1 and 5. In Experiment I, the settings for the climate chamber were the same as those for germination and growing of the seedlings (Figure 5).

day parameters: 22 °C [1]; 120 μM m^−2^·s^−1^; 16 h
night parameters: 16 °C; 0 μM m^−2^·s^−1^; 8 h

### 4.5. Experiment II: Heat Stress

Experiment II started on 10-day-old seedlings, and instrumental measurements were performed on days 1 and 5, and samples were also taken for analytical studies (Figure 5).

heat stress parameters: 35 °C [1]; 120 μM m^−2^·s^−1^; 8 h
day parameters: 28 °C; 0 μM m^−2^·s^−1^; 8 h
night parameters: 22 °C; 0 μM m^−2^·s^−1^; 8 h

### 4.6. Sampling

Sampling took place on the first day of the heat stress treatment and 4 days after. During a sampling day, first the biophoton emission measurement was taken, followed by SPAD measurement, and finally, the seedlings were cut into approx. 0.5 cm pieces, then mixed thoroughly, thus creating an average sample from which the FRAP and MDA measurements were conducted.

### 4.7. Determination of Fresh/Dry Weight Ratio (FDWR)

Determination of fresh/dry weight ratio: 1 g of leaves were measured by an Ohaus Discovery DV215CDM (Ohaus Corporation 1.800.672.7722 7 Campus Drive, Suite 310 Parsippany, NJ 07054 USA) analytical scale weighing up to 5 decimal places. The leaves were then dried out in a drying cabinet (Memmert SLE 600, Memmert GmbH + Co. KG, Aeussere Rittersbacher Strasse 38, D-91126 Schwabach, Germany) at 60 °C for 24 h. Then, the weight of the dried samples was recorded. Fresh/dry weight ratio was expressed as % and was calculated by (w_x_ − w_0_)/w_x_)·100, where w_x_ is the dry weight and w_0_ is the fresh weight.

### 4.8. Chlorophyll Content Estimation (SPAD Index Measurement)

The chlorophyll content of the leaves of wheat seedlings was estimated via SPAD index, by reading 10 individual points on 10 seedlings of each treatment with SPAD-502 (Soil Plant Analysis Development–SPAD-502; Konica Minolta Sensing Inc., Osaka, Japan) equipment. 

### 4.9. Measurement of Lipid Oxidation 

Malondialdehyde (MDA) content was determined by the thiobarbituric acid reaction with some modifications of the original method [44]. Samples of 0.5 g were homogenized with 2 mL of 0.1% trichloroacetic acid in cold mortars from which 1.8 mL was transferred to Eppendorf tubes with automatic pipettes. To this solution, 40 μL of 20% butylated hydroxytoluene in absolute ethanol was added in order to stop further lipid oxidation. The solutions were vortexed for 15 s and centrifuged at 13,000 rpm for 10 min at 4 °C. From the clear supernatant, 0.25 mL was added to 1 mL of 20% TCA containing 0.5% TBA, gently mixed and briefly centrifuged for 5 s. The solutions were incubated in a water bath (Julabo ED-5M) for 30 min at 96 °C. The reactions were stopped by cooling the solutions immediately on ice followed by centrifugation at 10,000 rpm for 5 min. Absorbance at 532 and 600 nm was recorded using a SmartSpec™ Plus spectrophotometer, and MDA concentration was calculated by subtracting the non-specific absorption at 600 nm from the absorption at 532 nm using an absorbance coefficient of extinction, 156 mM^−1^ cm^−1^. The results were expressed as nmol g^−1^ in fresh weight (F.W.).

### 4.10. Ferric Reducing Antioxidant Power Assay

Total antioxidant activity was measured by the modified assay of ferric reducing antioxidant power (FRAP) of Benzie and Strain [45]. Samples of 0.5 g of fresh leaves were homogenized in leaves with a cool mortar and pestle using quartz sand and 4.5 mL 0.1 M phosphate buffer (pH 7.6, containing 0.1 mM EDTA). This suspension was centrifuged and refrigerated (Hettich, MIKRO 220R (Andreas Hettich GmbH & Co. KG Föhren str., 12 D-78532 Tutingen, Germany) at 10,000 rpm for 10 min [46]. The supernatant was used for the measurements. The constituents of the FRAP reagent were the following: acetate buffer (300 mM, pH 3.6), TPTZ (2, 4, 6-tripyridyl-s-triazine) 10 mM in 40 mM HCl and FeCl_3_ × 6H_2_O (20 mM). The working FRAP reagent was prepared by mixing acetate buffer, TPTZ and FeCl_3_ × 6H_2_O in the ratio of 10:1:1 at the time of use. Standard solution was 1000 µM ascorbic acid prepared freshly at the time of measurement. To 0.1 mL of the supernatant was added 2.9 mL of FRAP reagent in 5 mL screw cap centrifuge tubes, vortexed in a 37°C water bath (Julabo ED-5M, JULABO GmbH, 77960 Seelbach/Germany) for 4 min, and the absorbance was measured at 593 nm against a blank with a BIORAD SmartSpec™ Plus spectrophotometer (Bio-Rad Ltd., 1000 Alfred Nobel Drive, Hercules, CA 94547, USA). The FRAP values of the samples were determined in ascorbic acid (AA) equivalent (µg AA equivalent/g FW) based on the ascorbic acid calibration curve, as the averages of three independent measurements.

### 4.11. Biophoton Emission (BPE) Measurement 

BPE was measured using the NightShade LB 985 in vivo plant imaging system (Institute Bert-hold Technologies Bioanalytical Instruments, Calmbacher Strasse 22, D-75323 Bad Wildbad, Germany) with a sensitive thermoelectric Peltier-cooled, slow-scan NighOwlcam CCD device installed. The instrument was controlled by IndiGo ™ 2.0.5.0 software. 

The seedlings to be tested were placed in the dark chamber of the apparatus for 20 min before the start of the measurement per treatment, i.e., dark-adapted, to avoid chlorophyll autofluorescence.

Pixel intensity peaks could be detected by the camera and saved by the software. The settings were constant; the changes in the obtained relative pixel intensities reflected the amount of photon emission generated by the given treatment and the different temperature conditions, the value of which was converted to count per second (cps) using the analysis of IndiGo ™ 2.0.5.0 software. The integration time was set to 60 s with 4 × 4 pixel linking, and the measurement was taken for 5 min. During image acquisition, ‘background correction’ and attenuation of cosmic radiation were performed simultaneously to eliminate high-intensity gamma radiation.

### 4.12. Statistical Analysis

The measurements were repeated three times. The resulting dataset was analyzed by using one-way ANOVA (*p* < 0.05) coupled with Duncan post hoc test to prove the differences between groups. Additionally, two-way ANOVA (*p* < 0.05) was applied to study the mutual effect of parameters on the independent variable using the IBM SPSS 20.0 software.

## 5. Conclusions

In our studies, we found that the harmful effects of free radicals during heat stress can be alleviated by using a biostimulant. We also showed that the biophoton emission measurement is suitable for detecting the effects of heat stress and the heat stress-reducing effect of the biostimulant on wheat seedlings. Furthermore, we found that biostimulatory pretreated plants showed higher antioxidant capacity and dry matter content during heat stress compared to the untreated group, as well as a significant decrease in biophoton release and lipid oxidation, which may encourage the development of more efficient and cost-effective biostimulant use practices and the use of biostimulants for seed priming purposes.

## Data Availability

The data presented in this study are available on request from the corresponding author.
